# Use of REGEN-COV in children after heart transplantation for treatment and post-exposure prophylaxis of COVID-19

**DOI:** 10.1017/S1047951122002190

**Published:** 2022-07-07

**Authors:** Dipankar Gupta, Stephanie A. Clifford, Frederick J. Fricker

**Affiliations:** 1 Congenital Heart Center, Shands Children’s Hospital, University of Florida, Gainesville, FL, USA; 2 Department of Pediatrics, College of Medicine, University of Florida, Gainesville, FL, USA; 3 College of Nursing, University of Florida, Gainesville, FL, USA

**Keywords:** COVID-19, REGEN-COV, paediatric heart transplant

## Abstract

COVID-19 pandemic continues to evolve and new variants like Delta and Omicron have been discovered. REGEN-COV is a recombinant human monoclonal antibody to the spike protein of SARS-CoV-2 which received emergency use authorisation for treatment and post-exposure prophylaxis in patients with high risk of progression to severe disease. We review our experience with use of REGEN-COV in paediatric heart transplant patients.

The World Health Organization declared coronavirus disease (COVID-19) secondary to SARS-CoV-2 infection a pandemic on 11 March, 2020. Since then multiple variants including Delta-B.1.617.2 and Omicron-B.1.1.529 have been discovered with variable rates of infection in children. In presence of pre-existing comorbidities including diabetes mellitus, obesity, and cardiovascular disease, COVID-19 is associated with substantial risk of complications including mortality. Limited data is available about the risk and severity of COVID-19 in solid organ transplant patients. Sharma et al. reported a more frequent infection in African American population and higher use of renal replacement therapy in solid organ transplant patients, but the risk of severe disease and death was not higher than age-matched controls.^1^ A recent analysis from Pediatric Heart Transplant Society revealed that COVID-19 infection in paediatric heart transplant candidates and recipients leads to a higher rate of hospitalisation when compared to the general paediatric population. Despite higher rates of hospitalisation, the long-term sequelae and mortality remain low in these patients.^2^ Similarly, Goss et al. reported no difference in survival in paediatric solid organ transplant patients when compared to immunocompetent patients.^3^ Conversely, there have been reports of higher short-term mortality in patients with solid organ transplant as well.^4^ Certainly, these reports provide conflicting data, and the results are influenced by the time of infection in context of evolution of the pandemic, geographical area, type of variant, and other clinical features.

The available medical therapies for the management of COVID-19 infection continue to evolve with better understanding of pathophysiology and ongoing research. One such therapy, REGEN-COV, is a recombinant human monoclonal antibody to the spike protein of SARS-CoV-2, comprising Casirivimab and Indevimab. REGEN-COV initially received emergency use authorisation by the US FDA for use as post-exposure prophylaxis for COVID-19 in adult and children (≥12 years and ≥40 kg) at considerable risk for progression to severe COVID-19. It was also authorised for the treatment of mild-to-moderate COVID-19 with positive RT-PCR, in patients at considerable risk for progression to severe COVID-19. With increasing prevalence of Omicron variant and ineffectiveness of REGEN-COV against this variant, FDA revoked the emergency use authorisation stating that REGEN-COV is no longer authorised for treatment or post-exposure prophylaxis in geographic regions where infection is likely to have been caused by a non-susceptible SARS-CoV-2 variant based on available information such as variant susceptibility to this drug and regional variant frequency. We sought to review our experience with use of REGEN-COV, while it was authorised for use for treatment and post-exposure prophylaxis of COVID-19 in paediatric heart transplant patients.

A retrospective chart review approved by the Institutional Review Board at the University of Florida with waiver of consent was performed to identify patients who received REGEN-COV for post-exposure prophylaxis or as treatment to prevent progression to severe COVID-19. Detailed demographic and clinical data were collected from the electronic medical record. At our centre, six children received REGEN-COV from August to September 2021 during the Delta variant surge. Of these, four received REGEN-COV for the treatment after a positive RT-PCR and two received as post-exposure prophylaxis with a negative RT-PCR. In our cohort, the median age was 16.5 years (range: 15–19) and median time from transplant was 29 months (range: 2–140). There were equal number of males and females. Of those positive for COVID-19, three patients demonstrated mild symptoms (two respiratory and one gastrointestinal) and one patient was asymptomatic. Both patients who received post-exposure prophylaxis were asymptomatic; however, they were <1 year from transplant. All patients received REGEN-COV within 5 days of testing positive or exposure and tolerated it without any side effects. Patients who received it for post-exposure prophylaxis continued to remain RT-PCR negative and did not develop any symptoms. On evaluation of myocardial function, none of the patients demonstrated any changes on their echocardiogram after the infection and tolerated the infusion without any complications. Table 1 provides further details of immunosuppression at the time of COVID-19 infection or exposure, panel reactive antibodies, and other comorbidities. Immunosuppression regimens were not modified for any of the patients during or after the infection.

**Table 1.** Demographic information on patients who received monoclonal antibody infusion for COVID-19 infection or post-exposure prophylaxis

Patient ID	Age (Years)	Time from Tx (months)	Diagnosis	Immunosuppression medications	COVID-19 RT-PCR	Hospitalization/oxygen requirement	Comorbidities	PRA/DSAs
1	15	60	CHD	MMF, TAC, RAPA, PDN	Positive	None/None	Familial hypercholestrolemia	Wk positive/Pos
2	16	31	CHD	MMF, TAC	Positive	None/None	None	Neg/Neg
3	15	27	CHD	MMF, TAC, RAPA	Positive	None/None	CVID-SQIG dependent	Neg/Neg
4	19	140	CHD	TAC, PDN	Positive	None/None	Bronchiectasis, CVID-SQIG dependent	Neg/Neg
5	17	8	CMY	MMF, RAPA, Letermovir, PDN, Rituximab, Bortezomib	Negative (PEP)	None/None	Ganciclovir resistant CMV viremia	Mod-strong/Pos
6	18	2	CHD	MMF, TAC, PDN	Negative (PEP)	None/None	Obesity, Diabetes	Pos/Pos

CMY = cardiomyopathy; CVID = common variable immunodeficiency; DSA = donor-specific antibody; MMF = mycophenolate; PDN = prednisone; PRA = panel reactive antibody; RAPA = sirolimus, SQIG = subcutaneous immunoglobulin; TAC = tacrolimus,

COVID-19 continues to be a challenge, and the uncertainty with emergence of new variants is an ongoing issue especially for immunocompromised complex children with additional comorbidities. REGEN-COV has been found to be not effective for the Omicron variant and is no longer available for use if the infection is likely to have been caused by a non-susceptible SARS-CoV-2 variant based on available information such as variant susceptibility and regional variant frequency. Recently, Spinner et al. evaluated the SARS-CoV-2 anti-spike IgG antibodies after SARS-CoV-2 vaccination in paediatric heart transplant patients. They concluded that “a significant proportion of paediatric heart transplant recipients (30%) have no detectable antibody response after SARS-CoV-2 vaccination.” These findings strongly suggest that the vaccination series should be completed prior to transplantation. Interestingly presence of neutropenia, diabetes mellitus, and previous use of rituximab were found to be associated with the absence of a detectable antibody.^5^

Currently Sotrovimab, a recombinant human IgG1κ monoclonal antibody that binds to a conserved epitope on the spike protein receptor-binding domain of SARS-CoV-2 received an emergency use authorisation by the US FDA for COVID-19 infection secondary to the Omicron variant. Due to inadequate immune response to vaccination, it is important to have therapies like these in our armamentarium to prevent progression to severe disease in solid organ transplant patients. As the pandemic evolves, the indications of previously and currently available therapies may continue to change.

In conclusion, REGEN-COV was tolerated without any complications in our cohort of paediatric heart transplant patients, and no modifications in immunosuppression were required.
